# Future human health research directions for the Canadian Northern Contaminants Program

**DOI:** 10.3402/ijch.v72i0.23049

**Published:** 2013-11-22

**Authors:** Shawn G. Donaldson, Meredith S. Curren, Bryan Adlard, Jonathan Provost, Tara Leech, Constantine Tikhonov, Mark Feeley, Scott Tomlinson, Russel Shearer

**Affiliations:** 1 Chemicals Surveillance Bureau, Healthy Environments and Consumer Safety Branch, Health Canada, Ottawa, ON, Canada; 2 EPH Surveillance and Risk Analysis, First Nations and Inuit Health Branch, Health Canada, Ottawa, ON, Canada; 3 Bureau of Chemical Safety, Food Directorate, Health Canada, Ottawa, ON, Canada; 4 Northern Science and Contaminants Research Directorate, Aboriginal Affairs and Northern Development Canada (AANDC), Gatineau, QC, Canada

**Keywords:** Northern Contaminants Program, POPs, metals, Arctic, human health

## Abstract

Studies conducted in the mid-1980s and early 1990s demonstrated that persistent organic pollutants (POPs) and metals were reaching the Arctic ecosystem at unexpectedly high levels, many of which had no Arctic or Canadian sources. Epidemiological and toxicological studies in Canada and in other countries have found that these contaminants may pose a risk to human health. The objective of this paper is to provide the foundation for the discussion on future northern human health research under the Northern Contaminants Program (NCP) in Canada. This short discussion of human health priorities will help guide a path forward for future northern human health research in Canada to address on-going and new health concerns related to contaminants exposure in the Canadian Arctic.

## Introduction

The Northern Contaminants Program (NCP) was established in 1991 in response to concerns about human exposure to elevated levels of contaminants in wildlife species that are important to the traditional diets of northern Aboriginal peoples (1). The NCP takes a partnership approach and is managed by a committee chaired by Aboriginal Affairs and Northern Development Canada (AANDC), which consists of four federal government departments (AANDC, Health Canada, Environment Canada, Fisheries and Oceans Canada), the territorial governments (Nunavut, Northwest Territories, the Yukon) and representatives of Northern Aboriginal organizations, including Inuit Tapiriit Kanatami (ITK), Inuit Circumpolar Council – Canada (ICC), Dene Nation and the Council of Yukon First Nations.

The results of NCP-funded human health research and monitoring form the basis for assessing risks to human health associated with contaminants in traditional/country foods. This information is used by regional health authorities to develop dietary advice for northerners, particularly those who are dependent on marine mammals and fish as an important part of their diets.

The NCP research program, combined with the voice of Northern Aboriginal organizations, has played an instrumental role in addressing the issue of northern contaminants internationally. The results provided by the NCP have contributed to the United Nations Economic Commission for Europe Convention on Long-Range Transboundary Air Pollution (UNECE-LRTAP) protocols on POPs and metals, the United Nations Environment Programme (UNEP) Stockholm Convention on POPs, and the 2013 Minamata negotiations on mercury. Internationally, the NCP data represents Canada's main contribution to the Arctic Monitoring and Assessment Programme (AMAP), which coordinates Arctic science activities in cooperation with the other circumpolar nations and their working groups under the Arctic Council. The NCP also contributes to Canada's involvement in new circumpolar Arctic science initiatives including the development of a Sustained Arctic Observing Network (SAON) as a legacy of International Polar Year (IPY) and Arctic Council climate change and contaminants issues.

## Future human health research

The NCP Human Health Technical Review Team, the NCP Management Committee and a number of Canadian Northern subject matter experts have identified priorities for future biomonitoring, health effects, and risk communication research.

### Human biomonitoring

Continued biomonitoring of POPs and metals is important to improve the understanding of contaminants in the North and to ensure that contaminant concentrations in the population and the associated human health risk continue to decline. Several emerging contaminants such as flame-retardants, perfluorinated compounds, and short-chained chlorinated paraffins have been detected in Arctic biota (2); however, there are limited available data on human exposures. The inclusion of new chemicals in future biomonitoring activities is recommended.

To place human chemical concentrations in a risk assessment context, biomonitoring equivalents (BEs) may be used during the evaluation of northern biomonitoring data. There is a need for tools to interpret biomonitoring data in a health risk context, which could include biomonitoring equivalents (3).

### Health effects

The NCP has supported a number of internationally recognized human health effects research studies. Future research could focus on the following topics in regions characterized by higher exposures and which are of concern to local health authorities:

- Child and maternal health effects (e.g., pregnancy complications, maternal illnesses during pregnancy, duration of gestation and foetal growth);
- Physical, motor, cognitive, behavioural, and emotional development from infancy to childhood, adolescence, and adulthood;
- Diabetes, metabolic syndrome, and cardiovascular disease;
- Other chronic diseases (e.g., allergy, asthma, Alzheimer's and Parkinson's disease, cancer); and
- Immune system function.

Health effects studies in those areas of greatest interest should take into account co-exposure to mixtures of chemicals and factors likely to modulate vulnerability of exposed individuals.

### Risk communication

Territorial and regional health agencies have the authority and responsibility to provide health advice or advisories about contaminants to northern residents. The most successful risk communication processes involve a variety of regional experts as well as the public in the communication process. Increased knowledge of local risk perception and how people make dietary choices is necessary to support the decision-making process.

Additional research should involve evaluating the effectiveness of previously published risk communications (in collaboration with appropriate public health authorities) including assessing factors influencing the success and effectiveness of communication activities and determining the optimal context for the delivery (2).

## Strategic research design

The focus of the human health component for the next phase of the NCP should be on the continued development of integrated regional multidisciplinary studies, which would include human biomonitoring, health effects research, risk communication and the assessment of contaminant sources. This would include developing standard study protocols to ensure comparability of results across geographic locations and time.

## Conclusion

The NCP's flexible, collaborative, and adaptable framework has generated internationally recognized science that is used to manage the Arctic contaminants issue at many levels. The NCP has been successful due to its promotion of responsible research, a partnership approach, investment in northern capacity building, and international collaboration and leadership. The future directions discussed here are in support of the NCP's objective to work towards reducing and, where possible, eliminating contaminants in traditional/country foods, while providing information that assists individuals and communities in making informed decisions about their food use.
